# Thermal damage and excision time of micro and super pulsed diode lasers: A comparative ex vivo analysis

**DOI:** 10.1002/cre2.670

**Published:** 2022-10-11

**Authors:** Mariliza C. O. Prado, Ngozi N. Nwizu, Shalizeh A. Patel, Charles F. Streckfus, Denise Maria Zezell, Juliana Barros

**Affiliations:** ^1^ Laboratory of Biophotonics, Center for Lasers and Applications IPEN‐CNEN/SP São Paulo São Paulo Brazil; ^2^ Department of Diagnostic and Biomedical Sciences, University of Texas Health Science Center at Houston School of Dentistry Houston Texas USA; ^3^ Department of Restorative Dentistry & Prosthodontics, University of Texas Health Science Center at Houston School of Dentistry Houston Texas USA

**Keywords:** micro‐pulsed diode laser, oral biopsy, super‐pulsed diode laser, thermal damage

## Abstract

**Objectives:**

The primary aim of this ex vivo study was to evaluate thermal damage and cutting efficiency of micro and super pulsed diode lasers. The secondary aim was to suggest a guideline to perform simple surgical excisions adequate for histopathological evaluation.

**Material and Methods:**

Ten groups of 10 specimens of pig tongues were excised using a blade (G1), a micro pulsed (G2–G9), and a super pulsed diode (G10) lasers. Different output power, pulse duration, pulse interval, and duty cycle were tested. Quantitative measures of thermal damage and excision times were recorded. Statistical analysis was performed at a significance level of 5%.

**Results:**

The control group (G1) presented no thermal damage. Within the laser groups (G2–G10), no statistically significant differences in depth of thermal damage (µm) were noted. G3 showed significantly less area of thermal damage (mm^2^) when compared with G7 and G9 (*p* < .05). The median excision time of the control group and super pulsed diode laser group were significantly lower (*p* < .001) than the micro pulsed diode laser groups.

**Conclusions:**

The cutting efficiency of the super pulsed diode laser is comparable to traditional blade, and with appropriate parameters, these lasers can produce predictable surgical outcomes with less collateral damage.

## 1 INTRODUCTION

As standard of care, general dentists are to identify oral lesions, develop differential diagnoses, and provide appropriate treatment to patients. Currently, diode lasers (810–1064 nm) are used to perform various procedures such as ablation, incision, excision, and coagulation for intraoral surgeries. Dental diode lasers are minimally invasive and can become valuable in the treatment of soft tissue surgeries including biopsies. Hemostasis of surgical field, postoperative pain management, and faster healing rates are a few advantages of diode lasers well documented in the literature (Diamanti et al., 2002; Gontijo et al., 2005; Margarone et al., 1984; Wlodawsky & Strauss, 2004). As with any high‐intensity laser, diode lasers convert light into thermal energy. This interaction with the target tissue can produce different biological effects, ranging from coagulation to carbonization (Kende et al., 2011). Diode lasers’ unique affinity to interact with pigments within the targeted oral soft tissues, allows it to become a selective surgical tool. This leads to more controlled and less invasive surgical outcomes, reducing inflammation and pain and improving healing (Gold & Vilardi, 1994; Pick & Colvard, 1993; White et al., 1998).

However, laser‐tissue interaction produces some degree of tissue vaporization including a zone of thermal damage around the incision site. Thus, without the use of the appropriate parameters, diode lasers can induce excessive heat damage in the excised tissue, interfering with its histopathologic evaluation and ultimate diagnosis (Gold & Vilardi, 1994; Kende et al., 2011; Pick & Colvard, 1993; White et al., 1998; Wilder‐Smith et al., 1997).

High‐intensity lasers can emit light in two modes: continuous wave and pulsed. Diode lasers, in particular, have a constant emission of radiation. In the newer diode devices, a mechanical shutter gates the light transmission, shortening the pulse width allowing for thermal relaxation of the target tissue (Coluzzi & Convissar, 2011; Zezell et al., 2011). This feature allows the power to be delivered in repetitive pulse, ranging from milliseconds (ms) to microseconds (µs). It is important to note that a laser with a shorter pulse duration usually results in a lower temperature and thereby produces less carbonization of the tissue (Hobbs et al., 1987). The newest diode lasers are now equipped with super and micro pulsed technology, controlling its duty cycle, the fraction of time the laser is actively emitting energy (expressed in a percentage). This feature allows the laser to deliver high power in a short period of time, protecting the tissue from severe heat damage. It would be beneficial to carry out an ex vivo study to confirm such claims. Therefore, the primary aim of this ex vivo study was to evaluate the effects of different parameters on the integrity of collected specimens and the cutting efficiency of the two lasers’ pulse modalities. The secondary aim was to suggest a general guideline for clinicians to perform simple surgical excisions adequate for histopathological evaluation using diode lasers.

## 2 MATERIAL AND METHODS

**Figure 1:**
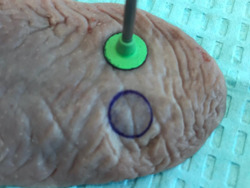
Picture of the pig tongue receiving a standardized 8 mm diameter delineation before laser excision.

**Figure 2:**
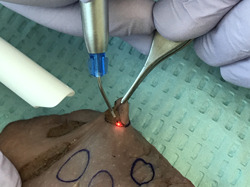
Picture of the excisional biopsy procedure on a pig tongue using a 300‐μm initiated fiber.

This study was IRB exempted; however, all the principles of safety, ethical, and professional conduct have been followed by the authors, including specimen disposal. Informed consent was not required for this study. Fresh pig tongues (within 24 h of slaughter) were used in this ex vivo study. The pig tongues were stored at a temperature of 2°C–4°C to prevent tissue degradation before the experiment (Gomez‐Santos et al., 2010). All excisions were made at room temperature between 22°C and 24°C (Melo et al., 2010). A 940 nm diode laser (Epic X™ ‐ micro pulsed diode laser and Epic Pro™ ‐ super pulsed diode laser, Biolase, Irvine, CA, USA) with 300 μm and 400 μm disposable‐initiated surgical tips, respectively, were used. One hundred standardized specimens with 8 mm diameter were excised from the dorsal portion of pig tongues (Figures 1 and 2) and randomly distributed into 10 groups (*n* = 10) as shown in Table 1. The control group (G1) biopsies were performed with conventional technique using 15C a scalpel blade. The specimens assigned to the micro pulsed diode laser groups (G2–G9) were excised at two average powers of 1.0 W and 1.5 W (Azma & Safavi, 2013; Gutiérrez‐Corrales et al., 2020; Romeo et al., 2014). Other variables within the laser unit were emission mode (continuous vs. pulsed), pulse duration (10 μs, 100 μs, and 1 ms), pulse interval (40 μs, 200 μs, and 1 ms), and duty cycle (20%, 33%, and 50%). The specimens assigned to the super pulsed diode laser group (G10) were removed at an average power of 3.2 W, peak power of 80 W, super pulse width of 10 µs–10 ms, pulse interval 0.01 ms–20 ms, and pulse repetition rate ranging up to 20 kHz (Romanos et al., 2016).

**Table 1:** Group distribution and diode lasers parameters

	**Group**	**Pulse mode**	**Duty cycle**	**Pulse Repetition rate (Hz)**	**Power at display (W)**	**Power measured (W)**	**Power density calculated (W/cm** ^**2**^ **)**	**Peak power at display (W)**	**Pulse width**	**Pulse Interval (off time)**
Scalpel	G1 (*n* = 10)	Control	n/a	n/a	n/a	n/a	n/a	n/a	n/a	n/a
Epic X™ ‐ micro pulsed diode laser (940 nm)	G2 (*n* = 10)	CW	n/a	20,000	1.2	1	1415.4	1.2	n/a	n/a
	G3 (*n* = 10)			20,000	1.8	1.5	2123.1	1.8		
	G4 (*n* = 10)	CP0	20%	20,000	1.2	1	1415.4	6	10 μs	40μs
	G5 (*n* = 10)			20,000	1.8	1.5	2123.1	9		
	G6 (*n* = 10)	CP1	33%	20,000	1.2	1	1415.4	3.6	100 μs	200 μs
	G7 (*n* = 10)			20,000	1.8	1.5	2123.1	5.4		
	G8 (*n* = 10)	CP2	50%	20,000	1.2	1	1415.4	2.4	1 ms	1 ms
	G9 (*n* = 10)			20,000	1.8	1.5	2123.1	3.6		
Epic Pro™ ‐ super pulsed diode laser (940 nm)	G10 (*n* = 10)	STP	n/a	40	n/a	3.2	2547.7	80	10 μs–100 ms	0.01 ms–20 ms

**Figure 3:**
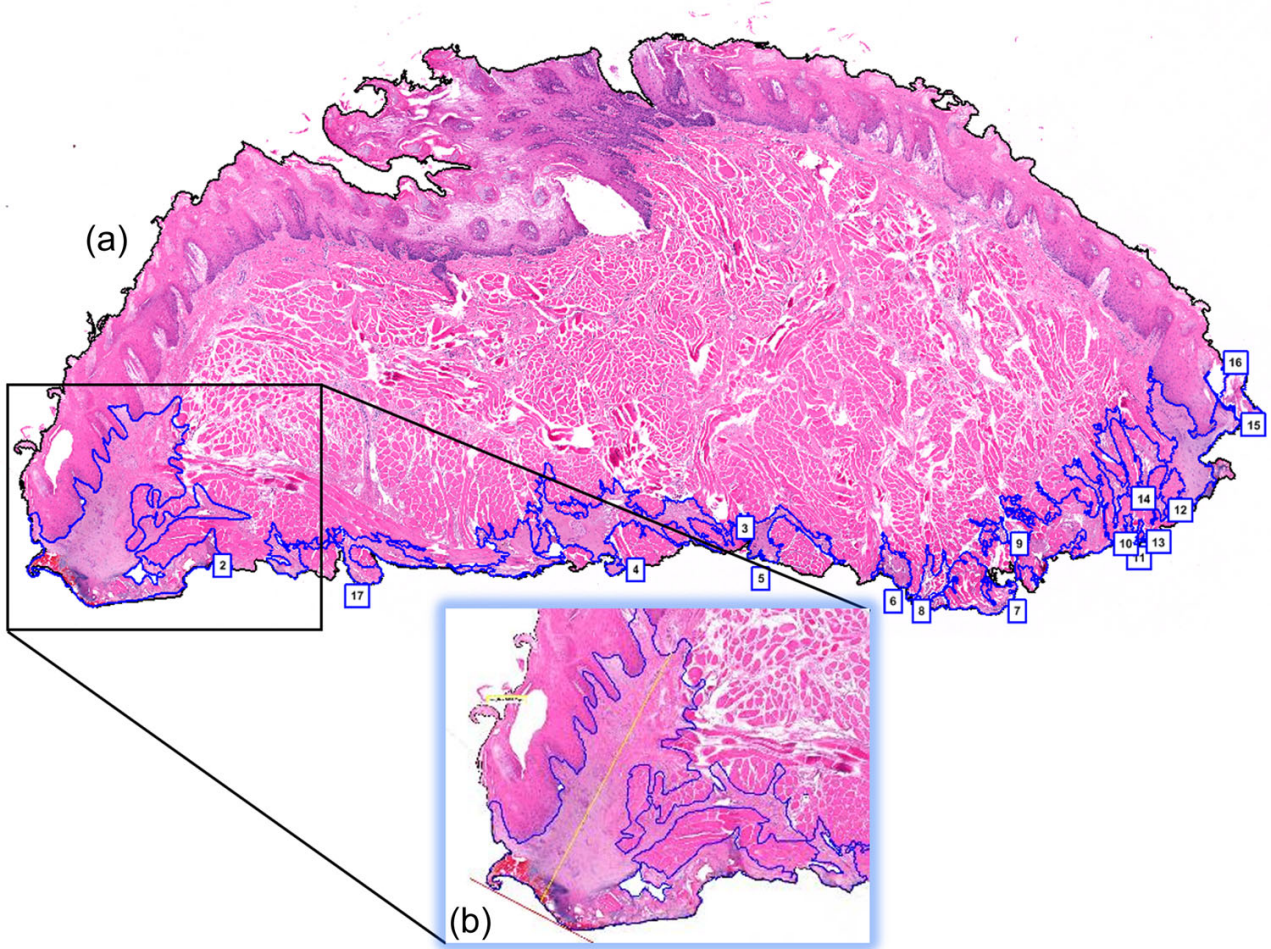
Representative images of the two thermal damage measurements (area and depth). (a) Measurement of the total area of damage is delineated in blue and calculated in millimeter square (mm^2^). (b) Measurement of depth of thermal damage is outlined in yellow in micrometers (µm).

The output power was checked before the start of each surgical procedure using a power meter (PM600 Power Meter, Molectron Detector Inc, Portland, OR, USA). The surgical time of each excision was recorded from the time of the laser application on the tissue to the complete removal of the sample. The specimens were fixed in 10% buffered formalin solution, cut into three equal‐sized slices, and embedded in paraffin blocks, according to conventional histological processing methods (Feldman & Wolfe, 2014; Fox et al., 1985). Serial sectioning of 4 μm thickness was performed for subsequent staining with hematoxylin and eosin (H&E). The histopathological images were evaluated under vertical light microscopy (Eclipse 80i, Nikon Corporation, Tokyo, Japan) and were captured with Andor Zyla 5.5 sCMOS (Andor Technology Ltd. Company, Belfast, UK). Each slide was observed at 40X magnification using the Nikon Eclipse 80i Advanced Research Microscope software (Nikon Instruments Inc., Melville, NY, USA). Both area (mm^2^) and depth (µm) of tissue thermal damage were recorded. The total area of thermal damage was quantified by calculating the difference between the total area of each specimen and its total area of damage, visually detected by the microscope (Figure 3a). Next, the area with the most thermal damage on each specimen was identified. A virtual 90° line from the base of the specimen was drawn within the most heat‐affected area (Figure 3b).

Statistical analyzes were performed using the IBM® SPSS software package, V20.0 **(**IBM Corp., Armonk, NY, USA) with a significance level of 5%. Shapiro–Wilk normality test was performed. The observed distributions were asymmetric and described by medians and quartiles, minimum and maximum values. Kruskal–Wallis nonparametric tests were applied to compare the study groups in relation to thermal damage areas, thermal damage depths, and excision times. Differences were reported using Dunn's multiple comparison tests with Bonferroni correction. The correlation between depth of thermal damage and percentage of area with thermal damage was evaluated by Spearman's rank correlation test.

## 3 RESULTS

### 3.1 Area of thermal damage

The control group (G1) presented no thermal damage. In experimental groups (G2–G10), the observed total area of thermal damage (mm^2^) was lower in the G3 group (1.5 W, CW) with a median of 0.91 mm^2^ and *p* < .05 (Table 2). Applying multiple comparison tests and controlling at the global significance level, the median of the damaged area of the G3 and G10 groups were lower than the adjusted medians of G7 (1.5 W, 33% duty cycle) and G9 (1.5 W, 50% duty cycle) groups. No statistically significant differences were observed in the other groups.

**Table 2:** Total area of thermal damage (mm^2^) of the specimens according to the study group

Group	Minimum	First quartile	Median	Third quartile	Maximum	*n*
**G2**	0.46	1.03	1.09	1.54	1.98	10
**G3**	0.40	0.53	0.91*	1.11	2.60	10
**G4**	0.71	0.91	1.22	1.86	2.99	10
**G5**	0.93	1.26	1.37	1.66	3.93	10
**G6**	0.34	1.20	1.53	1.72	1.90	10
**G7**	1.32	1.52	1.93*	2.45	3.71	10
**G8**	0.73	1.09	1.50	2.42	3.37	9
**G9**	0.77	1.40	1.97*	3.50	3.97	10
**G10**	0.57	0.90	1.08	1.74	2.76	9

* Kruskal–Wallis test: *p* = .009. G3 was significantly different when compared to G7 and G9; G1, the control group was excluded from the thermal damage evaluation as no laser was used.

### 3.2 Depth of thermal damage

The control group (G1) presented no thermal damage. In experimental groups (G2–G10), the depth of thermal damage (μm) in the specimens indicated no evidence of statistical differences amongst the groups (*p* = .125) (Table 3).

**Table 3:** Depth of thermal damage (μm) of the specimens according to the study group

Group	Minimum	First quartile	Median	Third quartile	Maximum	*n*
**G2**	317.6	489.4	606.4	858.1	1114.7	10
**G3**	468.0	574.5	655.8	742.8	863.2	10
**G4**	462.0	566.8	720.4	779.9	918.3	10
**G5**	429.7	482.5	789.6	1080.0	1206.2	10
**G6**	465.1	590.8	669.3	960.4	1129.8	10
**G7**	599.5	839.8	1024.9	1393.5	1555.0	10
**G8**	437.3	510.5	666.4	849.7	1397.9	9
**G9**	319.0	584.6	831.1	1057.5	1414.0	10
**G10**	470.6	530.5	778.8	947.1	1203.6	10

*Note*: Kruskal–Wallis test: *p* = .125. No statistical significance was detected amongst all groups. G1, the control group was excluded from the thermal damage evaluation as no laser was used.

### 3.3 Correlation of area and depth of thermal damage

**Figure 4:**
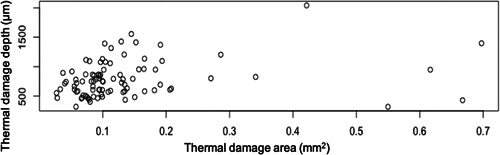
This graph illustrates the positive correlation between depth (µm) and area (mm^2^) caused by thermal damage.

The total area of thermal damage (mm^2^) ranges from 0.91 to 1.97 mm^2^ (Table 2) and the depth of thermal damage ranges from 606 to 1024 μm (Table 3). A positive correlation was noted between the depth (μm) and area (mm^2^) when evaluating thermal damage (Figure 4).

### 3.4 Excision time

It is noteworthy that the median excision time of the super pulsed diode laser group (G10) is comparable to the control group (G1–scalpel). Excision time medians in G1 and G10 were significantly lower (*p* < .001) than the medians of the micro pulsed laser groups (G2–G9) (Table 4).

**Table 4:** Excision time(s) of specimen according to study group

Group	Minimum	First quartile	Median	Third quartile	Maximum	*n*
**G1**	38	42	50*	68	108	10
**G2**	187	201	238	270	350	10
**G3**	122	179	194	227	244	10
**G4**	97	147	197	213	317	10
**G5**	110	122	161	194	258	10
**G6**	116	130	155	190	293	10
**G7**	131	142	155	158	306	9
**G8**	106	138	193	248	309	9
**G9**	110	122	142	150	205	9
**G10**	62	66	69*	75	83	10

* Kruskal–Wallis test: *p* < .001. Note that G1 (scalpel) and G10 (super pulsed diode laser) were statistically significantly different from all the other groups (*p* < .05).

## 4 DISCUSSION

Several studies have demonstrated that CO_2_, Er:YAG, Nd:YAG, and diode lasers can be used in oral soft tissue biopsies without causing significant thermal damage and hindering the histopathological diagnosis (Fox et al., 1985; Merigo et al., 2013; Romeo et al., 2014). However, very few have evaluated and compared variables such as thermal damage and excision times produced by micro and super pulsed diode lasers, the most common surgical lasers in clinical dentistry (Angiero et al., 2012; Belikov et al., 2015; Fornaini et al., 2016; Monteiro et al., 2019; Romeo et al., 2014). To bridge this gap, the present study evaluated two diode lasers with different combinations of power, duty cycle, and pulse duration. The findings of this study support the view that different parameters can variously affect soft tissue responses, such as thermal damage and cutting efficiency. In addition, this study showed a positive correlation between area and depth of tissue affected when measuring overall thermal damage. Thus, our discussion reflects findings on both methodologies, when referencing thermal damage.

This present study noted that the micro pulsed diode laser G3 (CW, 1.5 W) produced less total area of thermal damage (mm^2^) when compared to all groups. The groups with the greatest area of thermal damage (mm^2^) were G9 (1.5 W, 50% duty cycle) and G7 (1.5 W, 33% duty cycle), while the least damage was identified as G3 (CW, 1.5 W) followed by G10 (super pulsed laser, 3.2 W). These observations can partially be explained by a phenomenon called the “hot tip” effect in combination with the laser's pulse duration. The hot tip effect is caused when a target tissue absorbs the laser energy, leading to protein denaturation and carbonization of the tissue. This leads to a carbonized build‐up at the tip of the fiber, which continues to overheat, into several hundred degrees, and promotes tissue cutting, coagulation, and possible thermal damage (Verdaasdonk & Swol, 1997). Therefore, it is recommended to constantly stop and remove this carbonized build‐up debris from the laser tip. Though removing this aggregate of debris is needed, the cleaned tip has now a reduced ability to superficially interact with the tissue. This allows the laser light to penetrate deeper into the target tissue. As this carbonization build‐up and cleaning cycle continues, a fluctuation in the depth of thermal penetration and cutting efficiency within the same surgical site is noted.

In addition to the variance in this heat penetration, the laser pulse duration is another major factor that can negatively impact tissue response to uncontrolled thermal damage. Laser pulse duration is referred to the time tissue is exposed to laser energy. The longer the time, the more laser‐tissue interaction is observed (Belikov et al., 2015; Spencer et al., 1998). Additionally, the amount of power (peak vs. average) can dictate the degree of interaction within the surgical site. Peak power is the maximum optical power a laser can produce to interact with the target tissue within a specific time frame. Average power is the power transferred to the target tissue per unit of time. Regardless of what power parameter is utilized, it is important to balance thermal effects within a tissue by allowing the thermal energy to dissipate. This time of cooling is referred to as thermal relaxation (Stuart Nelson et al., 1995). A proper laser protocol must encourage the reduction of heat‐generated collateral damage by balancing both power and pulse duration. Pulse duration greater than 1μs has displayed noticeable thermal effects on oral soft tissue (Niemz, 2007). In the present study, groups G7 and G9 with larger pulse duration (100 μs and 1 ms) and a high peak power (5.4 W and 3.6 W) resulted in a greater area of heat transmission within the specimens. In contrast, the super pulse diode laser (G10) had a pulse duration longer than 1 μs (10 μs–10 ms) and high peak power (80 W), but caused less thermal penetration. This can be explained by the new tip technology within this super pulsed diode laser (Epic Pro™), where a controlled thermal feedback mechanism is installed to read tissue's temperature and adjust its level at the fiber tip during excision time. This advanced system aims to prevent overheating of the tissue and improve cutting efficiency (Romanos et al., 2016). Our study findings showed good congruence with those of Wilder‐Smith et al. (1997); they observed that incisions created with a super pulsed mode CO_2_ laser (pulse duration of 300 μs, peak power 60–100 W, and average power of 0.7–1.2 W) showed a significant reduction in collateral thermal damage when compared with continuous mode (Wilder‐Smith et al., 1997). Our results also corroborated those of Romanos’ findings, as the time duration for removing biopsied tissues obtained with the super pulsed diode laser (G10), was significantly faster than other groups (Romanos et al., 2016). In addition, it was noted that the cutting efficiency of the super pulsed diode laser was comparable to a scalpel.

The present study utilized pig tongues as they are histologically and physiologically similar to human tongues (Azevedo et al., 2016; Cercadillo‐Ibarguren et al., 2010; Romeo et al., 2010). The investigators selected an 8 mm diameter as the standardized specimen size for two main reasons. Laser surgery is not recommended for any lesion less than 5 mm in size due to the high probability of heat‐induced artifacts interfering with microscopical evaluation (Angiero et al., 2012; Vescovi et al., 2010). Second, 8 mm represents the average size of most simple excisional biopsies of common oral lesions +1 mm allowance of a halo of healthy peripheral tissue for proper histological analysis. Besides, the larger specimen size is easier to handle in an ex‐vivo setting. For example, fibromas which are the most frequent (53.3%) benign tumors of the oral cavity vary in size between 5 and 8 mm (Allon et al., 2014; Gonsalves et al., 2007; Torres‐Domingo et al., 2008). The most common locations for these slowly growing lesions are the buccal mucosa along the occlusal line and the tongue (Gonsalves et al., 2007; Torres‐Domingo et al., 2008). Treatment is surgical excision and recurrence is rare. Per literature, it is advised when using laser as a surgical tool, to leave a halo of 0.5–1 mm of healthy tissue peripheral to pathology. This preserves the morphological and structural characteristics of the collected specimen allowing for a readable and reliable diagnosis (Romeo et al., 2014). According to this study, the deepest area of thermal damage within the specimen ranged in on average from 606 to 1024 μm supporting Romeo et al suggestion of leaving a band of healthy tissue peripheral to diseased margins to compensate for any heat‐induced artifacts (Romeo et al., 2010).

To the best of the authors’ knowledge, this is the first study to evaluate the effects of different parameters of micro and super pulsed diode lasers on the integrity of tissue specimen and its cutting efficiency. Therefore, it provides invaluable baseline information that would be useful for further studies. Diode lasers are the most common surgical lasers utilized by general dentists, thus their proper use, including appropriate biopsy parameters should be standardized. This study provides useful information that can guide clinicians in the laser excision of simple surgical biopsies, improve surgical outcomes and patient safety.

For study limitations, the use of pig tongue mucosa, despite the similarities, is not human tongue mucosa, the authors recognize there is a possibility that there might be some variations in the results obtained using actual human tongue mucosa. Furthermore, this study had several limitations regarding the comparison between the new super pulsed laser and the micro pulsed laser, since in the latter it is not possible to control the peak power and pulse width, as it presents an intrinsic variable, unlike the super pulsed laser. Another limitation of this study is the attainment of an exact specimen size in a reproducible manner. Even though a diameter of 8 mm was set, all measurements were not always exactly precise because laser cutting of the tissue was done manually and may have introduced the possibility of small random errors in mm^2^. To overcome this limitation, however, the investigators tested two methods of measuring thermal damage (depth and area). After statistical analysis, it was concluded that both measurements correlated positively and can be applied independently or collectively.

Future clinical studies are needed to confirm this study's finding of high peak power and long pulse duration of micro pulsed laser can generate greater thermal damage in comparison to super pulsed lasers.

## 5 CONCLUSION

In this study, the investigators aimed to (1) evaluate the thermal effects of different parameters on the integrity of collected specimens excised by micro and super pulsed diode lasers, (2) compare the cutting efficiency of the two diode laser systems, and (3) suggest a general guideline for clinicians to perform simple surgical excisions adequate for histopathological evaluation. The results of the study confirmed the use of diode lasers as a suitable surgical tool for excision of simple benign tumors when appropriate parameters are used. A minimum of 1 mm perimeter of healthy margins is recommended for appropriate microscopic analysis of the tissue specimen. This “safe zone” will be sufficient to withstand collateral damage caused by laser heat under the parameters described in this study. In addition, the super pulsed diode laser with the new thermal feedback system demonstrated a more efficient cutting ability than the micro pulsed laser and was comparable to the scalpel. However, not many clinicians are able to replace their existing lasers with the latest technology as they become available. Thus, it is relevant to apply knowledge gained from this *ex vivo* study to suggest general guidelines for best clinical outcomes. Per results of this study, clinicians using:

- *conventional diode lasers* may consider a continuous mode of emission rather than a 50% duty cycle;
- *micro pulsed diode laser* may select the shorter pulsed duration parameter offered within the laser or continuous mode of emission;
- *super pulsed diode laser* may select the lowest cutting power parameter offered within this new generation of lasers.

The above guideline can reduce heat‐generated collateral damage allowing for proper histopathological evaluation of biopsied tissues. Nevertheless, there is a need for further randomized clinical trials comparing these types of diode lasers to precisely establish their differences and to recommend parameters for the best results in a clinical setting.

## AUTHOR CONTRIBUTIONS

Dr. Mariliza Prado has contributed to the material preparation, data collection, and analysis, original draft of the manuscript. Dr. Ngozi Nwizu has contributed to the study conception and design, data analysis, review, and editing of the manuscript. Dr. Shalizeh Patel has contributed to the material preparation, data analysis, review, and editing of the manuscript. Dr. Charles Streckfus has contributed to the statistical analysis and review of the manuscript. Dr. Denise Zezell has contributed to the study conception and design, review, and editing of the manuscript. Dr. Juliana Barros has contributed to the study conception and design, material preparation, data analysis, review, and editing of the manuscript. All authors read and approved the final manuscript.

## CONFLICTS OF INTEREST

The authors have received a grant for laser consumable supplies from Biolase, Inc. Irvine, USA that partially supported this study.

## ETHICS STATEMENT

This study does not contain any involving human participants or animals. For this type of ex vivo study, no ethical committee was needed, the current study is in accordance with the ethical standards of The University of Texas Health Science Center at Houston.

## Data Availability

The data that support the findings of this study are available from the corresponding author upon reasonable request.
